# Short-term prolactin administration causes expressible galactorrhea but does not affect bone turnover: pilot data for a new lactation agent

**DOI:** 10.1186/1746-4358-2-10

**Published:** 2007-07-24

**Authors:** Gabrielle Page-Wilson, Patricia C Smith, Corrine K Welt

**Affiliations:** 1 Reproductive Endocrine Unit, Massachusetts General Hospital, Boston, USA; 2 Harvard Medical School, Boston, USA

## Abstract

**Background:**

Medications used to augment lactation increase prolactin secretion but can have intolerable side effects. We examined the biological activity of recombinant human prolactin (r-hPRL) as preliminary data for its use to augment lactation.

**Methods:**

Healthy, non-postpartum women (n = 21) with regular menstrual cycles underwent a seven day randomized, double-blind, placebo-controlled trial of r-hPRL. Expressible galactorrhea, markers of bone turnover, calcium homeostasis and gonadal function were measured and side effects recorded.

**Results:**

Prolactin levels increased during r-hPRL administration (20.0 ± 2.8 to 231.7 ± 48.9 μg/L at 6 hours; p < 0.05). Five of nine participants who received r-hPRL developed expressible galactorrhea (p < 0.001). Urinary deoxypyridinoline decreased and bone specific alkaline phosphatase increased in r-hPRL and placebo groups. Menstrual cycle lengths were not altered and side effects were similar between r-hPRL and placebo groups.

**Conclusion:**

In summary, r-hPRL can cause expressible galactorrhea. Seven days of r-hPRL administration does not adversely affect bone turnover or menstrual cyclicity. Thus, r-hPRL may be a viable option for short-term lactation augmentation.

**Trial registration:**

Clinical Trials.gov NCT00438490

## Background

Breastfeeding has many important health implications for mothers and infants [1]. However, there can be obstacles to breastfeeding even in the most motivated women. The prevalence of lactation insufficiency may be as high as 15% in newly lactating women [2]. The rate of lactation insufficiency is even higher in mothers of premature infants who must pump breast milk to feed their babies in neonatal intensive care units [3]. The causes of lactation insufficiency are multifactorial and include poor sucking, structural breast abnormalities and infrequent feedings by the infant. They also include poor milk production and poor letdown [2].

A subset of mothers with poor milk production has insufficient prolactin secretion, although the absolute prolactin levels required for adequate lactation and the number of women affected is unknown. Prolactin is critical to breast milk production based on absence of lactation in the absence of prolactin [4] and evidence that bromocriptine abolishes lactation at many stages, even late postpartum when basal prolactin levels have reached the normal range [5]. The majority of studies demonstrate a relationship between prolactin levels and milk volume [6-10], with low basal and suckling stimulated prolactin concentrations in women with the poorest lactation [9]. Agents that increase endogenous prolactin, such as metoclopramide and domperidone, have been used in a 7–10 day course to increase breast milk supply in mothers with lactation insufficiency [11-14]. However, these medications can be associated with side effects such as drowsiness and depression and alternative therapies are needed [12,15,16]. Recombinant human prolactin (r-hPRL) is a potentially new therapy that is available for investigational use; however, its biological activity and side effect profile have not been examined.

As a consequence of the large calcium requirements of lactation, markers of bone formation and resorption are increased [17] and bone density declines at trabecular and cortical sites [18]. Women with pathological hyperprolactinemia exhibit reduced radial and vertebral body bone mineral density compared to controls [19]. It has been suggested that the decrease in bone density observed during lactation and in hyperprolactinemic women is largely due to the hypoestrogenemia that can accompany hyperprolactinemia [20,21]. However, the bone loss during lactation exceeds that in women with GnRH agonist-induced hypoestrogenemia [22]. Similarly, women with pathological hyperprolactinemia and amenorrhea exhibit lower radial bone mineral content than amenorrheic women with normal prolactin levels [23]. Further, prolactin levels correlate inversely with bone density and markers of bone formation and directly with markers of bone resorption [24]. Finally, prolactin receptors have been identified on osteoblasts [25]. Taken together, these data suggest that prolactin may have a direct effect on bone turnover in addition to effects mediated by estrogen suppression. These potential prolactin effects on bone must be identified in estrogen replete women to isolate effects related directly to prolactin and not the concomitant estrogen deficiency in hyperprolactinemic states.

Lactation is also associated with amenorrhea. The etiology of lactational amenorrhea may include the suckling stimulus, energy deficit related to the increased catabolic demands of lactation, increased sensitivity to estradiol feedback and elevated prolactin levels [26]. We have previously shown that prolactin has direct effects on GnRH secretion in the absence of changes in menstrual cycle length when given for a seven day course [27]. The absence of a direct effect of r-hPRL on menstrual cycle length needs confirmation.

The goal of this study was to determine whether r-hPRL was biologically active and to examine potential side effects from the use of r-hPRL on bone turnover and menstrual cyclicity, since prolactin may have direct effects on these parameters as outlined above. We chose not to study lactating women for these pilot studies because the enormous changes in bone turnover and the hypoestrogenic state that accompany lactation could not be separated from the effect of prolactin administration itself. Therefore, we treated regularly cycling, estrogen replete, non-postpartum women with prolactin at doses targeted to achieve prolactin levels during lactation [9,28-30]. Using estrogen replete, non-lactating women allowed us to isolate the direct effects of r-hPRL from effects related to the postpartum state and the concomitant estrogen deficiency.

## Methods

A total of twenty-one healthy women aged 21 – 38 years (28.4 ± 5.4 years, mean ± SD) were studied during the years 2002 and 2003 in a randomized, double blind, placebo-controlled trial of recombinant human prolactin administration (r-hPRL; Genzyme Corporation, Cambridge, MA [31]). All participants had a history of regular 25 – 35 day menstrual cycles with ovulation documented by a luteal phase progesterone level. All participants had normal prolactin and thyroid stimulating hormone (TSH) levels, renal function, hemoglobin ≥ 110 g/L, body mass index (BMI) 17.5 – 30.4 kg/m^2^, and no evidence of androgen excess, expressible or spontaneous galactorrhea or breast masses. Participants were non-smokers, had no history of osteoporosis, alcoholism or medical problems, and used no medications known to affect bone turnover and no hormonal medications for at least three months. The study protocol was approved by the Massachusetts General Hospital (MGH) Human Research Committee and all participants gave written informed consent before participation.

Participants were studied in the follicular phase of the menstrual cycle, starting between days 1 and 8 after menses. Before the first injection, participants underwent a breast exam to document the absence of expressible galactorrhea and collected a 24 hour urine sample for calcium, creatinine, phosphorus, deoxypyridinoline and N-telopeptide. At the completion of the urine collection, participants had blood drawn for calcium, phosphorus, parathyroid hormone (PTH), parathyroid related protein (PTH-rP), 1,25-(OH)_2 _vitamin D, albumin, bone specific alkaline phosphatase, estradiol, luteinizing hormone (LH) and follicle stimulating hormone (FSH). Participants also kept a 48-hour food record, which was used to match calcium, phosphorus, sodium and protein consumed during the 48-hour period surrounding the final r-hPRL injection.

The participants were randomized by the research pharmacy using a random number generator to receive a subcutaneous (SC) r-hPRL or placebo injection for seven days. To achieve prolactin levels in the range of those measured in postpartum women, i.e. 100–250 μg/L [9,28-30], the first subject was treated with 300 μg/kg r-hPRL based on pharmacokinetic data from the manufacturer (Genzyme Corporation). However, prolactin levels in the first subject reached 900.6 μg/L using this protocol and the dose was decreased to 60 μg/kg r-hPRL for subsequent participants. The data from the first subject was not included in the mean prolactin level calculations. Blood was drawn for prolactin levels at baseline and 2, 4, and 6 hours after the injection and vital signs were monitored during the 3 hours immediately following the injection. A transvaginal or transabdominal ultrasound was performed and the maximum diameter of all follicles ≥ 10 mm was recorded.

Participants either returned daily to the GCRC or self-administered 60 μg/kg r-hPRL or placebo injections for six additional days. Blood was drawn for estradiol, LH and FSH levels before each daily injection. On the day before the final injection, participants began a second 48-hour food record to determine if dietary components matched those consumed in the first 48 hours of the study.

On the day of the final r-hPRL or placebo injection, blood was drawn as above and participants collected a second 24-hour urine sample. The transvaginal or transabdominal ultrasound was repeated to assess follicle growth. Twenty-four hours after the final r-hPRL or placebo injection, blood samples were drawn as on the day before the first injection and breasts were examined for the presence of expressible galactorrhea by palpation. Participants were asked to describe any side effects and adverse events were recorded. Participants returned 2–3 weeks later for a urine pregnancy test and an assessment of menstrual cycle length.

### Assays

Deoxypyridinoline (Dpd; Pyrilinks-D, Quidel, San Diego, CA), N-telopeptide (NTX; Osteomark, Ostex International, Inc., Seattle, WA), and bone specific alkaline phosphatase (BSAP; Alkphase-B, Quidel, San Diego, CA) were measured using enzyme-linked immunosorbent assays (ELISAs) according to the manufacturers' directions. For deoxypyridinoline the intra-assay coefficients of variation (CVs) were 8.5, 5.6, and 4.9% at concentrations of 10.6, 72.1, and 135 nM Dpd/mM creatinine (Cr), respectively, and the inter-assay CVs were 7.6 and 4.2% at concentrations of 18.0 and 99.9 nM Dpd/mM Cr. For N-telopeptide the intra-assay CVs were 8.6, 5.0, and 5.7% at concentrations of 155, 818, and 1630 nM bone collagen equivalents (BCE)/mM Cr, and the inter-assay CVs were 12.5 and 10.2% at concentrations of 407 and 1420 nM BCE/mM Cr. For BSAP, the intra-assay CVs were 3.3, 3.5, and 2.9% at concentrations of 12.3, 58.2, 96.7 U/L, and the inter-assay CVs were 3.7 and 6.5% at concentrations of 13.1 and 65.0 U/L. Serum prolactin, LH, FSH, and estradiol were measured using a 2-site monoclonal non-isotopic system according to the manufacturer's directions (Axsym, Abbott Laboratories, Abbott Park, IL). LH and FSH levels are expressed in IU per liter as equivalents of the Second International Reference Preparation 71/223 of human menopausal gonadotropins. The interassay CVs for the LH assay were 5.3, 5.5 and 7.4% at concentrations of 5.6, 26.2 and 69.0 IU/L. The interassay CVs for the FSH assay were 6.9, 7.1 and 6.3% at concentrations of 4.3, 35.4 and 79.5 IU/L. The CVs for the estradiol assay were 9.2, 5.4 and 9.6% at estradiol concentrations of 312, 1101, 2570 pmol/L. The CVs for the prolactin assay were 4.6, 4.5 and 5.2% for prolactin concentrations of 8, 20 and 40 μg/L. Intact PTH was measured using a two-site chemiluminescence immunoassay (Nichols Institute Diagnostics, San Clemente, CA). The sensitivity of the assay was 5 pg/mL and the interassay CVs were less than 5.5 and 6% at concentrations of 21.7 and 143.5 ng/L. PTHrP (1–86) was measured using a two-site immunoradiometric assay (Diagnostic Systems Laboratories Inc., Webster, Tx, USA). The sensitivity of the assay was 0.3 pM and the intra- and interassay CVs were 4.8% and 13.6%. 1,25-(OH)_2 _vitamin D was measured by radioimmunoassay (RIA; DiaSorin, Inc., Stillwater, MN). The interassay CVs were 14.6, 11.1 and 11.2% for quality control sera at low, medium and high concentrations. For all measurements, all samples for a given individual were run in the same assay.

### Data Analysis

Studying a total of 21 participants was expected to yield at least 8 participants who received r-hPRL, resulting in a greater than 90% chance of detecting galactorrhea at a 2-sided, 5% significance level if the rate of spontaneous or expressible galactorrhea in women with regular menstrual cycles is 1% [32]. Twenty-one participants also resulted in an 80% probability that the study would detect a significant difference in markers of bone turnover at a 2-sided, 5% significance level.

All data were normally distributed. A *z -*test was used to determine whether the rate of expressible galactorrhea exceeded 1%, the incidence in regularly cycling women [32]. Prolactin, gonadotropins, estradiol concentrations, markers of bone formation and resorption, calcium, phosphorus, albumin, PTH, PTHrP, 1,25-(OH)_2 _vitamin D and follicle size were analyzed using 2 way repeated measures ANOVA during r-hPRL or placebo administration and significant differences were further analyzed using a Tukey post hoc test. Calcium, phosphorus, sodium, vitamin D and macronutrient intake were compared before and after r-hPRL or placebo administration using paired t-tests. Results are expressed as mean ± SEM unless otherwise indicated. A *p *value of < 0.05 was considered significant.

## Results

**Figure 1:**
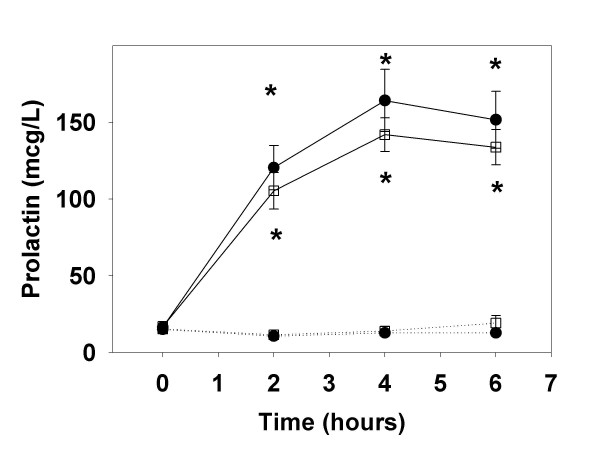
**Prolactin levels after r-hPRL or placebo injections**. Prolactin levels (mean ± SE) after administration of 60 μg/kg recombinant human prolactin (solid lines) or placebo (dotted lines) on the first (closed circles) and 7^th ^day of injections (open squares), over 6 hours. * designates significant differences in r-hPRL and placebo on the same day at p < 0.05.

In the r-hPRL treated group, prolactin levels increased from 16.6 ± 2.4 μg/L to a peak of 164.2 ± 20.3 μg/L 4 hours after the first 60 μg/kg r-hPRL injection (Figure 1). The prolactin level was unchanged at baseline before the seventh r-hPRL injection (16.9 ± 1.5 μg/L) and increased to a peak of 141.9 ± 10.9 μg/L four hours after the final r-hPRL injection. In the placebo group, baseline prolactin levels (15.0 ± 1.5 vs. 15.3 ± 2.0 μg/L; before and on the seventh day of placebo injections, respectively; p = NS) and prolactin levels over 6 hours (12.8 ± 1.8 vs. 19.1 ± 4.9 μg/L at 6 hours; p = NS; Figure 1) did not change after seven days of placebo injections, as expected.

None of the participants had expressible galactorrhea at baseline. During the seven days of r-hPRL administration, five of the nine participants experienced several drops of expressible galactorrhea (p < 0.001), and two of those five participants experienced breast tenderness or fullness. The breast secretions were milky white in color, bilateral and had no evidence of blood or abnormal coloration. Of the five participants who experienced expressible galactorrhea, two had been pregnant and one delivered a child 5 years before participating in the study. Of the four participants who did not develop expressible galactorrhea, three had been pregnant and two had delivered children 3 years and 7 years before participating in the study. There was no difference in age in the participants who experienced galactorrhea compared to those who had not (28.3 ± 5.1 vs. 29.8 ± 3.3 years, mean ± SD; p = 0.6; galactorrhea vs. no galactorrhea, respectively). None of the participants in the placebo group developed spontaneous or expressible galactorrhea. One subject receiving r-hPRL noted mild fatigue and moodiness. One subject had a tender lymph node in the groin area, thought to be unrelated to the r-hPRL. There were no other side effects in the r-hPRL treatment group. In the placebo group, side effects included diarrhea, nausea (n = 2), headache (n = 3), fatigue, fever and bruising at the blood drawing site.

Urinary deoxypyridinoline concentrations tended to decrease in the r-hPRL group (Table 1; p = 0.07) and did decrease in the group as a whole (8.2 ± 1.0 vs. 7.1 ± 0.8 nM Dpd/mM Cr; p < 0.05), but there were no significant changes in the placebo group. There were no changes in urinary N-telopeptide after seven days of r-hPRL or placebo treatment. Bone specific alkaline phosphatase increased during seven days of placebo treatment (p < 0.01) and in the group as a whole (16.1 ± 1.2 U/L vs. 17.7 ± 1.3 U/L; p < 0.01), but not during r-hPRL administration.

**Table 1:** Markers of bone turnover and calcium homeostasis during r-hPRL or placebo treatment

	**Placebo**		**r-hPRL**	
	**Before treatment**	**After treatment**	**Before treatment**	**After treatment**

**Deoxypyridinoline (nM/mM)**	7.2 ± 0.6	6.9 ± 0.7	9.5 ± 2.1	7.4 ± 1.8
**N-telopeptide (nM/mM Cr)**	45.3 ± 4.6	42.3 ± 5.6	35.6 ± 4.0	31.9 ± 4.1
**Bone specific alk phos (U/L)**	15.4 ± 1.3	17.5 ± 1.5*	17.0 ± 2.2	18.1 ± 2.5
**Calcium (mmol/L)**	2.3 ± 0.03	2.4 ± 0.03*	2.3 ± 0.03	2.4 ± 0.03*
**Albumin (g/L)**	40 ± 1	43 ± 1*	40 ± 1	42 ± 1
**Corrected calcium (mmol/L)**	2.4 ± 0.03	2.4 ± 0.03	2.4 ± 0.03	2.5 ± 0.05
**Phosphorus (mmol/L)**	1.2 ± 0.03	1.1 ± 0.03	1.0 ± 0.06	1.1 ± 0.06
**PTH (ng/L)**	45.7 ± 4.4	47.6 ± 4.5	46.8 ± 5.9	38.8 ± 6.2
**PTH-rP (pmol/L)**	0.33 ± 0.02	0.35 ± 0.04	0.30 ± 0.00	0.37 ± 0.05
**1,25-(OH)_2 _Vit D (pmol/L)**	102.2 ± 7.0	102.2 ± 6.7	115.4 ± 17.5	117.6 ± 25.2
**Urinary Ca/Cr**	0.11 ± 0.02	0.11 ± 0.02	0.10 ± 0.02	0.15 ± 0.02
**Urinary Phos/Cr**	0.61 ± 0.07	0.61 ± 0.08	0.60 ± 0.07	0.59 ± 0.05

All results: mean ± SE

* p < 0.05 within a group as indicated by the Tukey post hoc test.

Serum calcium concentrations increased significantly during seven days of r-hPRL and placebo administration, however, there was no significant effect of treatment on this increase (p = 0.74) and the change in calcium concentration was not different when corrected for albumin concentration (Corrected [Ca] = Measured total [Ca] + (0.8 × (4.5 - [alb])); Table 1). There were no changes in serum phosphorus, PTH, PTHrP, 1,25-(OH)_2 _vitamin D concentrations or in the urinary Ca/Cr and urinary phosphorus/Cr ratios after seven days of r-hPRL and placebo treatment (Table 1). There were also no changes in energy, macronutrient, calcium, phosphorus or sodium intake or in the Cr clearance in the 24 hours before r-hPRL or placebo injections began and in the final 48 hours of the injections (Table 2).

**Table 2:** Average daily energy, macronutrient, Vitamin D and mineral intake during r-hPRL or placebo administration

	**Placebo**		**r-hPRL**	
	**Before treatment**	**After treatment**	**Before treatment**	**After treatment**

**Energy (kcal)**	1963 ± 164	1929 ± 164	1670 ± 190	1606 ± 244
**Fat (g)**	65. ± 8.4	66.9 ± 6.7	58.3 ± 8.1	55.8 ± 11.4
**Protein (g)**	67.5 ± 7.5	71.8 ± 7.5	63.8 ± 7.1	62.9 ± 9.9
**Carbohydrate (g)**	283 ± 23	267 ± 25	225 ± 27	206 ± 33
**Vitamin D (μg)**	3.7 ± 0.9	4.1 ± 0.9	5.9 ± 3.8	2.9 ± 0.8
**Calcium (mg)**	849 ± 92	749 ± 113	1019 ± 162	1054 ± 210
**Phosphorus (mg)**	1166 ± 123	1131 ± 135	1093 ± 106	1029 ± 128
**Sodium (mg)**	3460 ± 436	3823 ± 497	2505 ± 222	2517 ± 328

All results: mean ± SE

**Figure 2:**
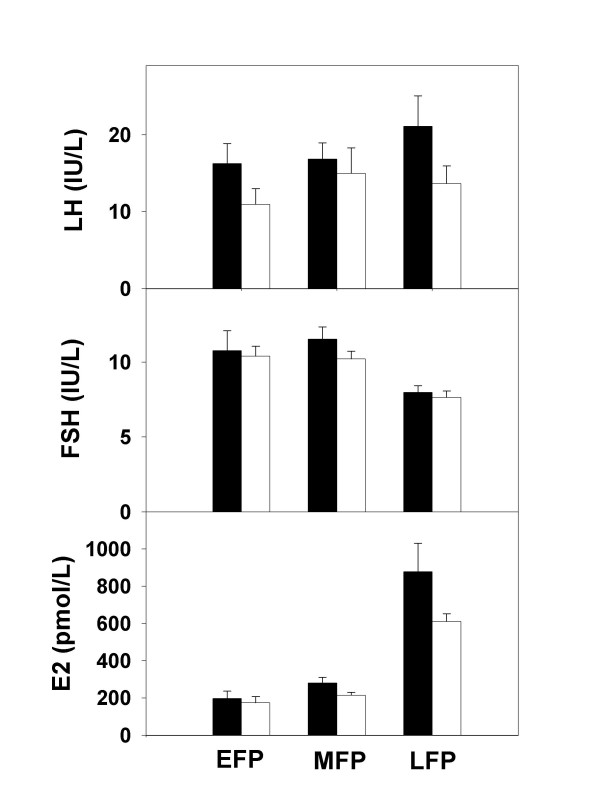
**Reproductive hormone concentrations during r-hPRL or placebo injections**. LH, FSH and estradiol (E2) concentrations (mean ± SE) in normal women during 7 days of treatment with placebo (n = 12; black bars) and r-hPRL (n = 9; white bars) in the early (EFP), mid (MFP) and late follicular phase (LFP). There were no differences in hormone concentrations at any cycle stage between the two groups.

Serum estradiol levels (217.3 ± 29.8 vs. 634.3 ± 175.1 and 186.1 ± 20.6 vs. 466.6 ± 72.4 pmol/L; before and after 7 days of placebo and r-hPRL, respectively; p < 0.05 for both) and follicle size (10.2 ± 0.8 vs. 15.9 ± 2.0 and 9.7 ± 0.8 vs. 15.6 ± 1.4 mm; p < 0.05 for both) increased after 7 days of placebo and r-hPRL treatment, as expected during the follicular phase of the menstrual cycle, and there was no difference between the two groups (Figure 2). There were no differences in LH and FSH between groups. There was no evidence of spotting or irregular menstrual bleeding during or after the study. Menstrual cycle length was similar during r-hPRL and placebo treatment (28.8 ± 1.0 vs. 30.1 ± 1.1 days; placebo vs. r-hPRL; p = 0.09).

## Discussion

Based on the overwhelming advantage of breast milk as an infant's source of nutrition coupled with a subset of women who have lactation insufficiency related to prolactin deficiency, additional medication to augment lactation without side effects is needed [3]. This study demonstrates the biological activity of r-hPRL in eliciting treatment-induced, expressible galactorrhea. In addition, r-hPRL induced no adverse changes in bone turnover when isolated from the hypoestrogenemia that occurs in physiological states of hyperprolactinemia [20,33,34]. There were also no changes in estradiol or menstrual cycle length in the current study or in our previous study in which r-hPRL was administered twice daily [27]. Finally, there were no significant adverse side effects reported. These data suggest that short-term use of recombinant human prolactin can produce expressible galactorrhea and is not detrimental to bone turnover or menstrual cyclicity.

In the current study, r-hPRL administration once daily for seven days was adequate to induce expressible galactorrhea, a time frame chosen based on studies of other lactation induction agents [11-14]. In a previous study in which r-hPRL was administered every 12 hours for seven days to examine its effect on GnRH secretion [27], four of six participants developed expressible galactorrhea, however, this study was not powered to examine efficacy. The pattern of hyperprolactinemia after r-hPRL administration does not perfectly replicate physiological or pathological hyperprolactinemia, however, it should be similar to the prolactin increase induced by metoclopramide. Studies are now ongoing to determine whether r-hPRL will augment milk production in postpartum mothers with lactation insufficiency and to determine the minimum dosing interval required. Further studies will determine whether r-hPRL can induce lactation in mothers with prolactin deficiency and absence of lactotrophs, such as women with a history of Sheehan's syndrome, and in adoptive mothers. Finally, although previous studies have suggested that the composition of milk in nonpuerperal women with induced lactation or hyperprolactinemia from medication, prolactinomas or hypothyroidism was similar to that of transitional or mature milk in composition [35-37], the milk composition will need to be examined in mothers who desire to lactate using r-hPRL long-term.

It is encouraging that r-hPRL did not increase markers of bone resorption or decrease bone specific alkaline phosphatase, a marker of bone formation, during short-term use for its potential development as a galactagogue. Nevertheless it is surprising. From the early follicular phase to the early luteal phase in non-lactating women markers of bone formation such as osteocalcin and bone specific alkaline phosphatase increase, as demonstrated in the current study and others [38-40], presumably in response to the increase in estradiol and possibly androgen levels. Markers of bone resorption such as urinary deoxypyridinoline, pyridinoline and N-telopeptide have been demonstrated to decrease [38], remain unchanged [41] or increase [40,41] from the early follicular phase through the early luteal phase of the menstrual cycle. In the current study, urinary deoxypyridinoline decreased across the follicular phase in the whole group of women. The findings in the current study are strengthened by the matched macro- and micronutrient intake at the time of the two evaluations. There was no independent effect of r-hPRL treatment on markers of bone formation or resorption, although there was a trend toward a decrease in urinary deoxypyridinoline in r-hPRL treated participants. Of note, short-term hyperprolactinemia did not disrupt the normal estradiol increase across the follicular phase of the menstrual cycle in our current or previous study using twice daily r-hPRL dosing, nor did a 12 hour r-hPRL infusion cause fluctuations in estradiol concentration [27]. Taken together, the absence of a change in bone formation markers and the trend toward a decrease in markers of bone resorption with short-term r-hPRL administration suggest that the increased bone resorption and formation observed in lactation [17] and the increased resorption and decreased formation in patients with hyperprolactinemia from prolactinomas [42] are associated with estrogen deficiency and changes in PTHrP that accompany these physiological states. Thus, the physiological effects of lactation would be expected to account for any changes in bone turnover during long-term r-hPRL administration.

The current study also examined the hormones controlling calcium homeostasis and determined that short-term hyperprolactinemia does not affect endogenous calcium concentrations. R-hPRL treatment did not decrease urinary calcium excretion, as had been demonstrated in animal models [43]. There was no increase in 1,25 (OH)_2 _vitamin D in women treated with r-hPRL for 7 days, suggesting that prolactin does not regulate 1-hydroxylation of vitamin D in women, consistent with previous observational studies [44-46]. There was also no increase in PTHrP during r-hPRL treatment, an observation that is surprising in light of the association between PTHrP and prolactin in lactating women and women with hyperprolactinemia [20,33,34] and interventional studies in animals [47,48]. Data demonstrating stimulation of PTH-rP in the suckled, but not the contralateral mammary gland provides evidence that a locally produced factor stimulates PTH-rP [49] and there is increasing evidence that extracellular calcium regulates PTHrP production via the calcium receptor in the lactating breast [50]. Nevertheless, it is possible that the duration of prolactin exposure in the current study was insufficient to increase PTHrP. Although the duration of hyperprolactinemia was sufficient to produce expressible galactorrhea in a majority of the participants, breast milk PTHrP levels increase with increasing duration of lactation [51-53] and women with longstanding prolactinomas and lactating women [20,33,34] are exposed to elevated prolactin levels for months, not days. Further, the volume of breast tissue may determine serum PTHrP levels [54]. Finally, a transient change in PTHrP during r-hPRL administration was not ruled out, although there was also no change in 24 hour urine concentrations of calcium and phosphorus, which would be expected to change with any transient change in PTHrP.

The current data also confirm and expand previous findings examining calcium changes across the menstrual cycle. When calcium was corrected for albumin, there was no change across the follicular phase, similar to previous studies [38,39,55]. There were also no changes in urinary calcium or phosphorus excretion, PTHrP or 1,25 (OH)_2 _vitamin D across the follicular phase in the current study or others [39]. Although PTH changes across the menstrual cycle have been more variable, demonstrating no change [39] a peak in the early luteal phase [38] or a decrease in the luteal phase [40], the current data demonstrate no change. Taken together, the data confirm and expand previous findings demonstrating that measurements of calcium homeostasis are not affected by the hormonal changes in the menstrual cycle.

## Conclusion

The data demonstrate that r-hPRL is biologically active, producing expressible galactorrhea in the majority of treated women with minimal side effects. Further, this randomized, placebo-controlled trial of r-hPRL administration for seven days isolated the effect of prolactin on bone from the effect of hypoestrogenemia and changes in the hormones regulating calcium homeostasis that can accompany hyperprolactinemia and lactation. The data demonstrate that r-hPRL does not have a detrimental effect on the markers of bone turnover. Further, short-term r-hPRL administration had no effect on estradiol or menstrual cycle length. Taken together, r-hPRL appears to have no direct detrimental effect on bone or menstrual cycle length during the short-term administration that would be used to augment lactation. Further studies are ongoing to examine short and long-term use of r-hPRL in lactating mothers.

## Competing interests

CKW received a sponsored research grant from Genzyme Corporation. Genzyme Corporation was not involved in the conception, study design, data analysis or writing of this manuscript.

## Authors' contributions

GPW carried out the protocol, collected and analyzed data and wrote the initial version of the manuscript. PS recruited participants, analyzed data, assisted with data interpretation and revised the manuscript. CW was responsible for study design, overseeing subject recruitment, data collection and analysis and revising the manuscript. All authors read and approved the final version of the manuscript.
